# Acupuncture for pain relief of women undergoing transvaginal oocyte retrieval: A meta analysis and systematic review protocol

**DOI:** 10.1097/MD.0000000000022383

**Published:** 2020-09-25

**Authors:** Xiao-Li Guo, Xiang Li, Wei Wei, Rong-Rong Wang, Fang Xiao, Li-Ying Liu, Jing Xu

**Affiliations:** Acupuncture and Tuina School, Chengdu University of Traditional Chinese Medicine, Chengdu, China.

**Keywords:** acupuncture, meta-analysis, pain relief, systematic review, transvaginal oocyte retrieval

## Abstract

**Background:**

Pain during oocyte retrieval, which can make the in-vitro fertilization process an unpleasant experience, is becoming a common problem. Although there are many analgesic methods available in the clinical setting, they are not therapeutically equivalent, and some are associated with varying adverse reactions. In recent years, acupuncture analgesia has been used in the perioperative period of oocyte retrieval because of its perceived efficacy and safety. The purpose of this systematic review and meta-analysis is to provide evidence that acupuncture is effective in the treatment of vaginal oocyte retrieval pain.

**Methods:**

Electronic searches of the following six databases will be conducted by two qualified reviewers: MEDLINE, EMBASE, China National Knowledge Infrastructure, Chinese Biomedical Medicine database, VIP database and Wanfang database. Three clinical trial registries will also be searched: World Health Organization International Clinical Trial Registry Platform, Chinese Clinical Trial Registry, Cochrane Central Register of Controlled Trials and ClinicalTrials.Gov. All searches will cover the period from inception of the database/registry to March 2020 and will be limited to publications in English and Chinese. Data identification, data selection, data extraction, and bias risk assessment will be conducted independently by3ν two or more qualified reviewers, including those who selected the studies. Visual analogue scale scores will be calculated as the primary outcome. Secondary outcomes will include results of other subjective pain rating scales, including Likert scales or other defined numerical or non-numerical scales, self-assessed by patients before, during, and after oocyte retrieval. We will use STATA software (Version 16) to perform meta-analyses, and the Grading of Recommendations, Assessment, Development and Evaluations framework to grade the quality of evidence. If quantitative analysis is not available, a systematic narrative synthesis will be provided.

**PROSPERO registration number:**

CRD42020170095.

## 1 Introduction

Oocyte retrieval is a crucial step of the in vitro fertilization and embryo transfer (IVF-ET) process. It is well known that ultrasound-guided vaginal oocyte retrieval is a routine procedure of IVF.^[1]^ The procedural time is generally short, however, mechanical stimulation of the needle through the vaginal wall and the ovaries can be painful for the patient.^[2]^ One study reported that 59.5% of patients experienced pain during oocyte retrieval.^[3]^ Other studies also confirmed that more than half of the women suffer pain during oocyte retrieval.^[4,5]^ Moreover, pelvic organs and blood vessels can be damaged by aspiration needles, and life-threatening complications related to infection or anesthesia may occur. Types of analgesia that have been used clinically for oocyte extraction include conscious sedation,^[6,7]^ general anesthesia,^[8]^ local injection as a paracervical block (PCB)^[9–14]^ and patient-controlled analgesia. PCB is the main analgesic method and has been widely used in some IVF units^[15,16]^ to relief pain. And a research has proved that the use of lignocaine in PCB did reach statistical significance.^[11]^ Although several meta-analyses and systematic reviews of analgesic interventions for oocyte retrieval have been published in recent years, there is currently no consensus on the optimal method.^[17,18]^ These analgesia methods can relieve pain, but may cause nausea, vomiting, dizziness, suppression of the nervous system, possible impairment of respiration or blood circulation and other adverse reactions.^[3]^

Nowadays, acupuncture is clinically accepted for analgesia.^[1,19–21]^ Acupuncture is widely used for pain relieve based on a principle of Traditional Chinese Medicine theory. Traditional Chinese Medicine theory states that the movement of qi and blood in the meridians is not smooth, block blocked, or the consumption of qi and blood is not nourish the meridians, which will form pain. The main pathologies of pain are “pain without general rule” and “pain without honor.” Acupuncture is also believed to nourish the meridians, regulate the flow of qi and blood to relieve pain.^[22]^ According to modern medical theory, acupuncture can relieve pain by stimulating receptors or nerve fibers in the tissue and producing a rhythmic discharge in the nerve fibers, leading to the release of endogenous neurotransmitters.^[23–29]^ Acupuncture also relieves pain by inhibiting visceral nociceptors, inflammatory cytokines, and by activating the central nervous system. Acupuncture analgesia for ultrasound-guided vaginal oocyte retrieval has been associated with few adverse reactions.^[1,24,30,31]^ A previous meta-analysis^[31]^ compared the pain relief effects of electro-acupuncture and conventional medical analgesic methods in 12 studies, concluding that there was no consensus on the best method for oocyte retrieval. However, the meta-analysis was limited by studies with insufficient sample sizes and low-quality literature, necessitating studies with larger sample sizes and a higher quality of evidence.

Therefore, the purpose of this study will be to review more new and larger randomized controlled trials (RCTs) of acupuncture analgesia compared with other analgesia methods during transvaginal oocyte retrieval, and to provide reliable clinical data on its safety and efficacy.

## 2 Methods

### 2.1 Registration

This protocol has been registered in PROSPERO (registration number: CRD42020170095). This system and meta-analysis will follow the Preferred Reporting Item for Systematic Evaluation and Meta-analysis (PRISMA)^[32]^ statement guidelines and the software STATA (version 16.0) will be used to construct the meta-analysis.

### 2.2 Eligibility criteria

#### 2.2.1 Types of study and participants

This review will only include RCTs (published and unpublished). Animal studies, case reports, commentaries, uncontrolled clinical trials and crossover trials will be excluded. Women undergoing transvaginal oocyte retrieval as part of a course of IVF treatments will be enrolled in the analysis.

#### 2.2.2 Types of interventions

The treatment group will receive acupuncture therapy. Traditional acupuncture, in which needles are inserted into definite acupoints, as well as modern acupuncture techniques such as transcutaneous electrical nerve stimulation (TENS), electro-acupuncture, point injection, acupressure, laser acupuncture, tap-pricking or cupping on pricked superficial blood vessels, will be included. The timing of acupuncture treatment will not be limited.

#### 2.2.3 Types of controls

All participants will receive PCB for analgesia during oocyte retrieval, with the following comparators:

- Acupuncture with PCB versus PCB alone;
- Acupuncture with PCB, versus invasive sham or minimal or noninvasive placebo acupuncture with PCB;
- Acupuncture with PCB, versus conscious sedation or drug analgesia with PCB;
- Acupuncture and conscious sedation or drug analgesia with PCB, versus conscious sedation or drug analgesia with PCB.

#### 2.2.4 Types of outcome measures

Participants must use the scale to self-assess pain at three time points: before, during and after the oocyte retrieval procedure. We define postoperative pain as pain measured over a period of time after oocyte retrieval.

- Primary outcome
  The visual analogue scale (VAS) will be the primary outcome for all studies.
- Secondary outcomes
  Studies must include a subjective pain rating scale such as a Likert scale or other defined numerical or non-numerical scale.

### 2.3 Search strategy

#### 2.3.1 Information sources

Six databases will be searched from inception to March 2020: MEDLINE, EMBASE, China National Knowledge Infrastructure, Chinese Biomedical Medicine database, VIP database and Wanfang database.

The main English-language retrieval terms will be as follows: “oocyte retrieval,” “transvaginal ultrasound oocyte retrieval,” “acupuncture,” “transcutaneous electrical nerve stimulation,” “electro-acupuncture,” “point injection,” “acupressure,” “laser acupuncture,” “tap-pricking,” and “cupping.”

Searching of the Chinese databases will be carried out using the corresponding search terms in Chinese.

#### 2.3.2 Other sources

The ongoing trials with unpublished data will be searched by following clinical trial registries: World Health Organization International Clinical Trial Registry Platform, Chinese Clinical Registry, Cochrane Central Register of Controlled Trials, and ClinicalTrials.gov. Manually retrieve and review a list of references to all possible published papers, including relevant systematic reviews, to identify any other trials.

## 3 Study selection and data collection

### 3.1 Data management

All of the documents will be under the charge of LYL. Each time one of the two independent reviewers has collected data from a paper, the original document will be screened and recorded. Once a source file has been entered, it will be recorded in the paper record sheet. Only study colleagues and other approved individuals will have access to the data used in this study.

### 3.2 Selection process

**Figure 1:**
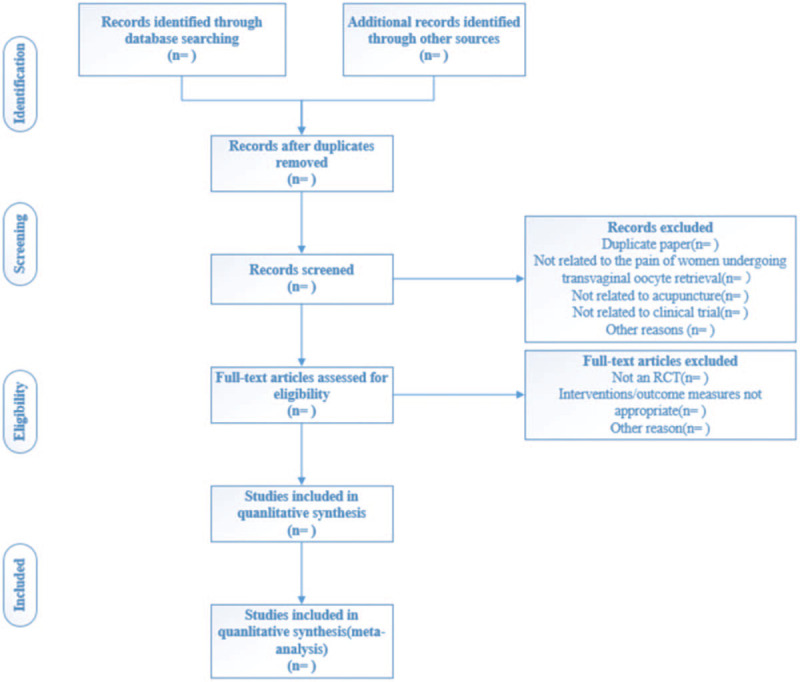
Flowchart of study selection. RCT = randomized controlled trial.

Two researchers (RRW and FX) will import the retrieved literature into Excel by title, abstract and full text, and review each article independently. The inclusion of studies for the review will be based on the PRISMA^[33]^ flow diagram (Fig. 1). If there is disagreement regarding selections by the two researchers, the disagreements will be resolved through discussion. If no agreement can be reached, a third researcher (LYL) will make the decision independently.

### 3.3 Data collection process

Similar to screening, data extraction will be carried out in duplicate by two independent reviewers (RRW and FX) to reduce bias and potential errors. Relevant information will then be entered into a unified data statistics table. Any disagreement will be resolved by discussion; if consensus cannot be reached, a third researcher (LYL) will make the final decision. All data will be cross-checked by FX and RRW, then transferred into STATA software (Version 16).

### 3.4 Data items

The information we plan to extract from each study includes general information (study title, first author, publication year and journal); characteristics (study object, diagnostic criteria, inclusion criteria, exclusion criteria, intervention details); outcome indicators and values. All included studies must meet the Standards for Reporting Interventions in Controlled Trials of Acupuncture (STRICTA)^[34]^ guidelines.

### 3.5 Missing data

If the full text of an article is not available or the information is incomplete, we will contact the original author for more information. If this method does not yield sufficient information, we will analyze the available data, report any missing data, and discuss the potential impact on the study.

### 3.6 Outcomes and prioritization

We will list and define each of the outcomes found in the retrieved articles and datasets. We will also prioritize these results based on the number of times each type of outcome appears in the collection of studies.

## 4 Data synthesis

### 4.1 Meta-analysis

STATA (Version 16) will be used to perform the meta-analysis. Dichotomous data will be represented as relative risk, and the Peto odds ratio method will be used to calculate 95% confidence intervals (CIs). The mean difference (MD) with 95% CI will express continuous variables. Standardized MDs and 95% CIs will be used for continuous variables when the units are different. Hedges’ g will be used to measure the statistical effect size after intervention as a continuous variable; accurate calculations will be used to calculate the bias-correction factor; and standard errors calibrated with Hedges and Olkin will be used to calculate the effect size. Two models will be used for this meta-analysis: random effects and fixed effects. The DerSimonian–Laird method and Mantel–Haenszel method will be applied in the random effects model, and the inverse-variance method will be applied in the fixed-effects model.

### 4.2 Assessment of heterogeneity

We will use the *I*-square (*I*^2^) and Cochran Q^[35,36]^ tests to evaluate the heterogeneity of articles. If *I*^2^ is <50% or the *P* value is >0.01, the heterogeneity is considered to be small. The fixed-effect model will be used to interpret low-heterogeneity results; the random-effect model will be used otherwise. Meta-regression will be adopted to investigate the potential sources of heterogeneity.

### 4.3 Additional analyses

If potential sources of heterogeneity are identified, such as types of acupuncture, control interventions, we will conduct sensitivity analysis, meta-regression analysis and subgroup analysis. If the sources of heterogeneity are insufficient, qualitative synthesis will be performed.

#### 4.3.1 Sensitivity analysis

Where appropriate, remove tests with a high risk of bias and then conduct a sensitivity analysis to determine whether the results of the evaluation are reliable. Independent studies will be categorized into groups according to research characteristics, such as statistical method, research methodology quality, and sample size. Combination analysis will be carried out using the Mantel–Haenszel method, followed by comparisons of the significant differences between each group and any combination effects.

#### 4.3.2 Subgroup analysis

In the case of sufficient data, subgroup analysis will be performed according to the following content:

(1) general information (eg region, age, baseline pain);
(2) acupuncture stimulation type (eg manual stimulation, electro-acupuncture, TENS, point injection, acupressure, laser acupuncture, tap-pricking or cupping on pricked superficial blood vessels);
(3) control intervention type (eg no treatment, sham acupuncture, drug anesthesia intervention).

#### 4.3.3 Meta-regression

If there is statistical or methodological heterogeneity, the meta-regression of the random-effect models will be used to explore the relationship between acupuncture therapy and oocyte retrieval pain. We will use the following factors as covariables for meta regression analysis:

(1) Participant characteristics: mean age and number of oocytes retrieved;
(2) Intervention characteristics: duration of acupuncture therapy and dose of PCB used for the pain;
(3) Reference characteristic: year of publication.

If the principle of this meta-regression technique cannot be proved, we will perform meta-regression analysis with limited covariates.

### 4.4 Assessment of reporting bias

If the meta-analysis contains ≥10 RCTS, we will use funnel plots and statistical tests (Egger's regression test^[37]^ and Begg's rank test) to examine the presence of publication bias. If the publication bias is non-ignorable, we will use the fill and trim method to correct the probable publication bias.

### 4.5 Assessment of risk of bias

Two reviewers (RRW and FX) will use the Cochrane Handbook for Systematic Reviews to evaluate the risk bias of each included trial.^[38]^ The following items will be included in risk of bias assessment categories, such as allocation concealment; random sequence generation; blinding of participants and outcome assessors; completeness of outcome data; selective outcome reporting and other biases. The risk of bias in each area will be divided into three grades: low risk, unclear risk, and high risk. The scores from all independent domains will be combined into an overall score that can be used to assess the risk of bias in the literature. If there is any disagreement, the decision will be made by the third researcher (LYL).

### 4.6 Systematic review

If quantitative synthesis is not appropriate, our team will provide a systematic narrative, in the form of text and tables, to explain and summarize the features and results of the included literature, and to explore and the relationships between the studies.

### 4.7 Quality of evidence

We will use the Grading of Recommendations Assessment Development and Evaluation^[39]^ (GRADE; Grade Pro version 3.6.1) to evaluate the results of the systematic evaluation based on the risk of bias, inconsistency, inaccuracy, publication bias and other factors. The quality of evidence will be divided into four grades: high, medium, low, and very low.

## 5 Discussion

It is currently difficult to assess the effectiveness and safety of acupuncture in women undergoing transvaginal oocyte retrieval because of the paucity of relevant, published RCTs. This review and meta-analysis will allow us to make adequate conclusions regarding the effectiveness of acupuncture for pain relief in women undergoing transvaginal oocyte retrieval. Further studies will be required to determine the optimal acupuncture treatment approach and investigate the neural pathways and molecular mechanisms of pain relief of this common procedure.

## Author contributions

Data curation: LYL, JX; Formal analysis: XLG, XL, LYL, JX; Investigation: XLG, WW; Methodology: RRW, FX; Project administration: JX; Resources: LYL; Software: RRW, FX, WW; Supervision: LYL; Writing – original draft: XLG, XL; Review & editing: XLG, XL, LYL, JX.

**Data curation:** Li-Ying Liu, Jing Xu.

**Formal analysis:** xiaoli guo, Xiang Li, Li-Ying Liu, Jing Xu.

**Investigation:** xiaoli guo, Wei Wei.

**Methodology:** Rong-Rong Wang, Fang Xiao.

**Project administration:** Jing Xu.

**Resources:** Li-Ying Liu.

**Software:** Wei Wei, Rong-Rong Wang, Fang Xiao.

**Supervision:** Li-Ying Liu.

**Writing – original draft:** xiaoli guo, Xiang Li.

**Writing – review & editing:** xiaoli guo, Xiang Li, Li-Ying Liu, Jing Xu.
